# Reduced Neurite Arborization in Primary Dopaminergic Neurons in Autism-Like Shank3B-Deficient Mice

**DOI:** 10.1007/s12035-024-04652-0

**Published:** 2024-12-09

**Authors:** Zuzana Bacova, Tomas Havranek, Denisa Mihalj, Veronika Borbelyova, Kristina Kostrubanicova, Michaela Kramarova, Daniela Ostatnikova, Jan Bakos

**Affiliations:** 1 Institute of Experimental Endocrinology, Biomedical Research Center, Slovak Academy of Sciences, Bratislava, Slovakia; 2 Institute of Anatomy, Faculty of Medicine, Comenius University in Bratislava, Bratislava, Slovakia; 3 Institute of Molecular Biomedicine, Faculty of Medicine, Comenius University in Bratislava, Bratislava, Slovakia; 4 Institute of Physiology, Faculty of Medicine, Comenius University in Bratislava, Bratislava, Slovakia

**Keywords:** Autism spectrum disorder, Dopaminergic neurons, *Shank3*, Neurite outgrowth

## Abstract

Despite many studies on dopamine changes in autism, specific alterations in midbrain dopamine neurons projecting to the striatum and cortex remain unclear. Mouse models with diverse SH3 domain and ankyrin repeat containing protein 3 (*Shank3*) deficiencies are used for investigating autistic symptoms and underlying neurobiological mechanisms. SHANK3 belongs to postsynaptic proteins crucial for synapse formation during development, and disruptions in SHANK3 structure could lead to impaired neurite outgrowth and altered dendritic arborization and morphology. Therefore, we aimed to investigate whether *Shank3* deficiency (*Shank3B*) leads to changes in the morphology of primary neuronal cell cultures from dopaminergic brain regions of neonatal mouse pups and whether it results in alterations in synaptic proteins in dopaminergic nerve pathway projection areas (striatum, frontal cortex). Significantly reduced neurite outgrowth was observed in primary dopaminergic neurons from the midbrain and striatum of *Shank3*-deficient compared to WT mice. A decrease in Synapsin I immunofluorescence signal in the cortical neurons isolated from *Shank3*-deficient mice was found, although neurite arborization changes were less severe. Importantly, the deficit in the length of the longest neurite was confirmed in primary cortical neurons isolated from *Shank3*-deficient mice. No changes in the gene expression of synaptic proteins were observed in the striatum and frontal cortex of *Shank3*-deficient mice, but an altered gene expression profile of dopaminergic receptors was found. These results show structural changes of dopaminergic neurons, which may explain autistic symptomatology in the used model and provide a basis for understanding the long-term development of autistic symptoms.

## Introduction

The pathogenesis of neurodevelopmental disorders involves structural changes in the shape and growth of neurons with a consequence on disrupted communication between specific brain regions [1, 2]. Several studies point to the fact that abnormalities in the development of dopaminergic nerve pathways could be a part of pathogenesis of autism conditions [3–5]. Although autistic symptoms, such as social communication abnormalities and repetitive behavior, are extensively studied at the behavioral level, the underlying cellular changes and neuronal growth alterations in dopaminergic brain areas remain unclear.

Individual dopaminergic neurons in the ventral tegmental area (VTA) projecting to the *nucleus accumbens* in the striatum (mesolimbic pathway) and the cortex (mesocortical pathway) are important for regulating social behavior [6, 7]. At the cellular level, changes in neurite outgrowth, arborization of dendrites, and the subsequent formation of synapses are particularly important for understanding potential structural alterations in autism. These changes can be observed and evaluated in vitro on isolated primary neuronal cell cultures. Tyrosine hydroxylase, the enzyme responsible for dopamine synthesis, is used to label specific dopaminergic neurons [8, 9].

Mouse models with diverse SH3 domain and ankyrin repeat containing protein 3 (*Shank3*) deficiencies are used for investigating autistic symptoms and underlying neurobiological mechanisms [10–12]. It is also known that SHANK3 belongs to postsynaptic proteins crucial for synapse formation during development [13, 14]. Moreover, mutations in gene encoding *Shank3* and/or disruptions in SHANK3 structure could lead to impaired neurite outgrowth and altered dendritic spine morphology contributing to the autistic phenotype [15–18]. Although data on dopaminergic pathways in the context of *Shank3* deficiency are scarce, increased dopamine receptor type 2 (D2) binding in the ventral striatum was found [3]. Another study reported that D2 receptor knockout results in autism-like behaviors [19]. Moreover, SHANK3 contributes to changes of VTA circuits and synapses on striatopallidal medium spiny neurons [9, 20]. However, it is not sufficiently known whether conditions of *Shank3* deficiency are directly associated with changes in morphology of dopaminergic neurons. So far, it is also unclear whether *Shank3* deficit could impact neurite outgrowth, synaptic proteins, or other structural changes in neurons within dopaminergic projection areas. Given that alterations in cytoskeletal rearrangement, neuritogenesis, and axon/dendrite elongation may represent the structural basis of autism pathology [16, 21, 22], it is crucial to identify specific changes in dopaminergic neurons.

Therefore, in this study, we aimed to investigate whether *Shank3* deficiency leads to changes in the morphology of primary neuronal cell cultures from dopaminergic brain regions of neonatal mouse pups and whether it results in alterations in synaptic proteins in dopaminergic nerve pathway projection areas (striatum, frontal cortex). Additionally, gene expression of dopaminergic receptors and selected dopamine metabolism enzymes were evaluated.

## Materials and Methods

### Animals

In these experiments, we used wild-type (WT) and homozygous knockout (*Shank3*^*−/−*^) mice, which were generated by mating of *Shank3B* heterozygous mice (B6.129-*Shank3*^tm2Gfng/J^) with PDZ depletion (exons 13 to 16) [10], obtained from the Jackson Laboratory (Stock No: 017688). Wild-type and *Shank3*^*−/−*^ mice (here also called *Shank3*-deficient mice) genotype status was confirmed via polymerase chain reaction (PCR) using DNA extracted from tail snips, following the Jackson Laboratory protocol. Following primers were used: common Fw: GAGACTGATCAGCGCAGTTG; WT Rv: TGACATAATCGCTGGCAAAG; SHANK3^−/−^ Rv: GCTATACGAAGTTATGTCGACTAGG. The resulting PCR products were analyzed by gel electrophoresis. Animals were kept on a standard water supply and pelleted diet ad libitum, group-housed (3–5 mice/cage) in temperature and light-controlled room (24 ± 2 °C, 12 h light/12 h dark). All experimental procedures were approved by the Ethical Committee of the Institute of Pathophysiology (07/2017/SKU11016), Comenius University, Bratislava, and have been conducted according to the European Union (EU) Directive 2010/63/EU and Slovak legislation.

### Preparation of Primary Neurons

Brains from WT and *Shank3*^−/−^ postnatal day 0 (P0) littermates’ mice (*n* = 8 (WT) or *n* = 6 (*Shank3*^−*/*−^)) were placed in ice-cold sterile Hank’s balanced salt solution, further supplemented with 100 U/ml penicillin and 100 U/ml streptomycin, 0.3 M Hepes (Sigma-Aldrich, Germany). Specific brain regions (frontal cortex, striatum, tegmentum) were individually collected under stereoscopic microscope (adapted according to Khazipov atlas [23]), with following coordinates for the frontal cortex (1.90 to 2.60 mm anterior to the bregma), the striatum (0.60 to 1.90 mm anterior to the bregma), and part of the ventral midbrain (tegmentum, − 2.80 to − 3.20 mm posterior to the bregma). Afterwards, tissues were enzymatically dissociated for 20 min at 37 °C (HBSS, 0.1% Trypsin, 0.1 mg/ml DNAse I) [12]. Cells from dissociated tissue were planted into 24-well plates with individual 12-mm round poly-d-lysin pre-coated coverslips (10 µg/ml; Sigma-Aldrich, Germany) and initially incubated for 3 h in RPMI medium containing 10% fetal bovine serum (both Sigma-Aldrich, Germany) under standard condition (37 °C and 5% CO_2_). After pre-incubation, RPMI medium was removed and exchanged for neuron-selective-growth medium (Neurobasal A; 100 U/ml penicillin; 100 U/ml streptomycin; 2 mM l-glutamine (all Gibco, USA) enriched with 2% B27 supplement (Invitrogen, USA)) for 5 days. Subsequently, on the 5th and 7th day in vitro (DIV5, DIV7), 50% of neuron-selective-growth medium volume was exchanged. For further morphological analysis, two coverslips per animal and specific brain region were used.

### Morphological Analysis

On the DIV9, stabilized culture of the primary neurons was inspected and fixed with 4% paraformaldehyde, pH 7.4 for 20 min at room temperature (RT). DIV9 was selected due to the intensive neurite outgrowth and synaptogenesis that occurs at this stage [24, 25]. Coverslips were gently washed two times with ice-cold PBS and blocked in normal goat serum (3% NGS in PBS) with the presence of 0.1% Triton X-100 for 30 min at RT. Specific proteins were detected with primary antibodies diluted in PBS (Table 1) for 120 min at RT. Afterwards, they were washed three times with ice-cold PBS and incubated with corresponding fluorescent secondary antibody diluted in PBS (Table 2) for 60 min at RT. Nuclei were marked by 300 nM DAPI (Thermo Fisher Scientific, Germany) for 1 min.

**Table 1:** Primary antibodies

*Name*	*Host species*	*Dilution*	*RRID*
*Anti-TH*	Rabbit	1:250	ab112; ABCAM, Germany
*Anti-Synapsin I*	Rabbit	1:500	SAB4502905; Sigma-Aldrich, Germany
*Anti-MAP2*	Mouse	1:2000	M4403; Sigma-Aldrich, Germany

**Table 2:** Fluorescent secondary antibodies

*Name*	*Host species*	*Dilution*	*RRID*
*Anti-mouse, Alexa Fluor 488*	Goat	1:500	A-11001; Thermo Fisher Scientific, Slovakia
*Anti-rabbit, Alexa Fluor 555*	Goat	1:500	A-21428; Thermo Fisher Scientific, Slovakia

Fluorescent signals from two coverslips per animal were collected using an Olympus BX63 at high magnification (× 20 objective) with constant detection limits and automatic deconvolution. At least ten areas per coverslip were evaluated by FIJI/IMAGE J software. Cells immunopositively stained for both MAP2 and TH were considered dopaminergic neurons. Arborization of dendritic tree was assessed by Neuroanatomy/Sholl analysis plugin. Detection of the number of intersecting dendrites with concentric circles in the 1-µm interval from the soma center up to 120 µm was performed. The longest neurite length was quantified from the edge of the nucleus to the apical end of the neurite by manual tracing. Synapsin I immunofluorescence signal was evaluated in three regions of interest (ROI) per cell, with the size of 20 × 5 µm, and data were represented as a percentage of the control group signal.

### Quantitative Real-time PCR

The relative expression mRNA levels isolated from the striatum (2.0 to 0.20 mm anterior to the bregma) and frontal cortex (4 to 2.60 mm anterior to the bregma) of WT (*n* = 8) or *Shank3*-deficient (*n* = 9) 21-day-old male mice were measured through real-time quantitative PCR (qRT-PCR). Total RNA was isolated by a phenol–chloroform method using TRI-reagent (MRC, Germany) according to the manufacturer’s protocol. Concentration and purity (260/280 and 260/230 ratio) of RNA were determined by the Nanodrop spectrophotometer (Thermo Fisher Scientific, Germany); however, RNA integrity was not assessed. Reverse transcription procedure was carried out using the High-Capacity cDNA Reverse Transcription Kit (Thermo Fisher Scientific, Germany). qRT-PCR was processed using Power SYBR® Green PCR Master Mix (Thermo Fisher Scientific, Germany) on QuantStudio 5 (Thermo Fisher Scientific, Germany) thermocycler. Relative gene expression levels were calculated by using the Livak method [26]. The 2^−ΔΔCt^ value of each sample was calculated using *Gapdh* as a reference control gene (primers sequences in Table 3).

**Table 3:** List of primer sequences used in this study. *Comt*, catechol-O-methyltransferase; *D1-D5*, dopamine receptors (1–5); *Gapdh*, glyceraldehyde 3-phosphate dehydrogenase; *Mao-a, b*, monoamine oxidase (A-B); *Nlgn 1–3*, neuroligin (1–3); *Psd95*, postsynaptic density protein 95; *Sv2a*, synaptic vesicle glycoprotein 2A

*Name*	*Primers*	*Gen Bank*	*References*
*Comt*	Fw: AGACCGCTACCTTCCAGACA	NM_001111063.1	[27]
	Rv: GTTCCCGGGACAATGACA		
*D1*	Fw: CCCCGAAAAGTGACTGAGATTGAC	NM_010076.3	[28]
	Rv: GCCGCTTGCTTTCCACCTG		
*D2*	Fw: ACGATGAGCCGCAGGAAGC	NM_010077.2	[28]
	Rv: GAAGGGCAGCCAGCAGATGA		
*D3*	Fw: CTCGGGGCCTTCATTGTCTG	NM_007877.2	[28]
	Rv: AGGAAGGCTTTGCGGAACTCTAT		
*D4*	Fw: TCGGGGCCTTCCTGGTGT	NM_007878.4	[28]
	Rv: TCCGCGTTGAAGATGGTGTAGAT		
*D5*	Fw: GGAGATCGCTGCTGCCTATGTC	NM_013503.4	[28]
	Rv: TGGGGGTGAGTGGTGAGATTTT		
*Gapdh*	Fw: CGGTGCTGAGTATGTCGTGGAGTC	NM_001289726.1	[29]
	Rv: CTTTTGGCTCCACCCTTCAAGTG		
*Mao-a*	Fw: CGGATATTCTCAGTCACCAATG	NM_173740.3	[27]
	Rv: ATTTGGCCAGAGCCACCTA		
*Mao-b*	Fw: CCAGAATCATCTCAACAACCAA	NM_172778.2	[27]
	Rv: TCACTTGACCAGATCCACCA		
*Nlgn1*	Fw: GGGGATGAGGTTCCCTATGT	NM_138666.4	[30]
	Rv: GGTTGGGTTTGGTATGGATG		
*Nlgn2*	Fw: AGGTGACCCTGAACGCATCA	NM_001364137.1	[30]
	Rv: AGCAGCCGCGTGTACTTGAG		
*Nlgn3*	Fw: CAGGCAACATGATTGATGGCAGTGT	NM_172932.4	[31]
	Rv: GAAGGCAATATTCTCACTCACCCAGCGA		
*Psd95*	Fw: TCTGTGCGAGAGGTAGCAGA	NM_007864.3	[32]
	Rv: AAGCACTCCGTGAACTCCTG		
*Sv2a*	Fw: GTCTGGTTTCCCGACATGAT	NM_022030.3	[33]
	Rv: CCTCAAACAGGGAATCCTCA		
*Synapsin I*	Fw: CACCGACTGGGCAAAATACT	NM_001110780.1	[33]
	Rv: TCCGAAGAACTTCCATGTCC		
*Synapsin II*	Fw: TGACAAATGCGTTCAGCTTC	NM_001111015.1	[33]
	Rv: TAGATGCCTTTCCTGGTTGG		
*Synaptophysin*	Fw: TCTTTGTCACCGTGGCTGTGTT	NM_009305.2	[32]
	Rv: TCCCTCAGTTCCTTGCATGTGT		

### Statistical Analysis

Statistical analyses were conducted using GraphPad Prism version 8.3.0. Outliers in all data were identified by the interquartile range method and excluded from further analysis. Gene expression and morphology data (longest neurite and Sholl analysis) were evaluated for normality distribution using the Shapiro–Wilk test. When normality was not confirmed, the Mann–Whitney test was applied to compare differences between groups (gene expression changes, longest neurites). For the Sholl analysis, a two-way ANOVA (factors: genotype and distance from nucleus) was employed, followed by Sidak’s post hoc test. To evaluate Synapsin I fluorescence intensity, the data were first tested for normality using the D’Agostino and Pearson test; since this passed, the comparisons between two groups were made using Student’s *t*-test. Results are presented as the mean ± SEM, with statistical significance set at *p* < 0.05.

## Results

### Shank3 Deficiency Leads to Morphological Changes in Dopaminergic Neurons and a Decrease in Synapsin I Levels in Cortical Neurons

**Fig. 1:**
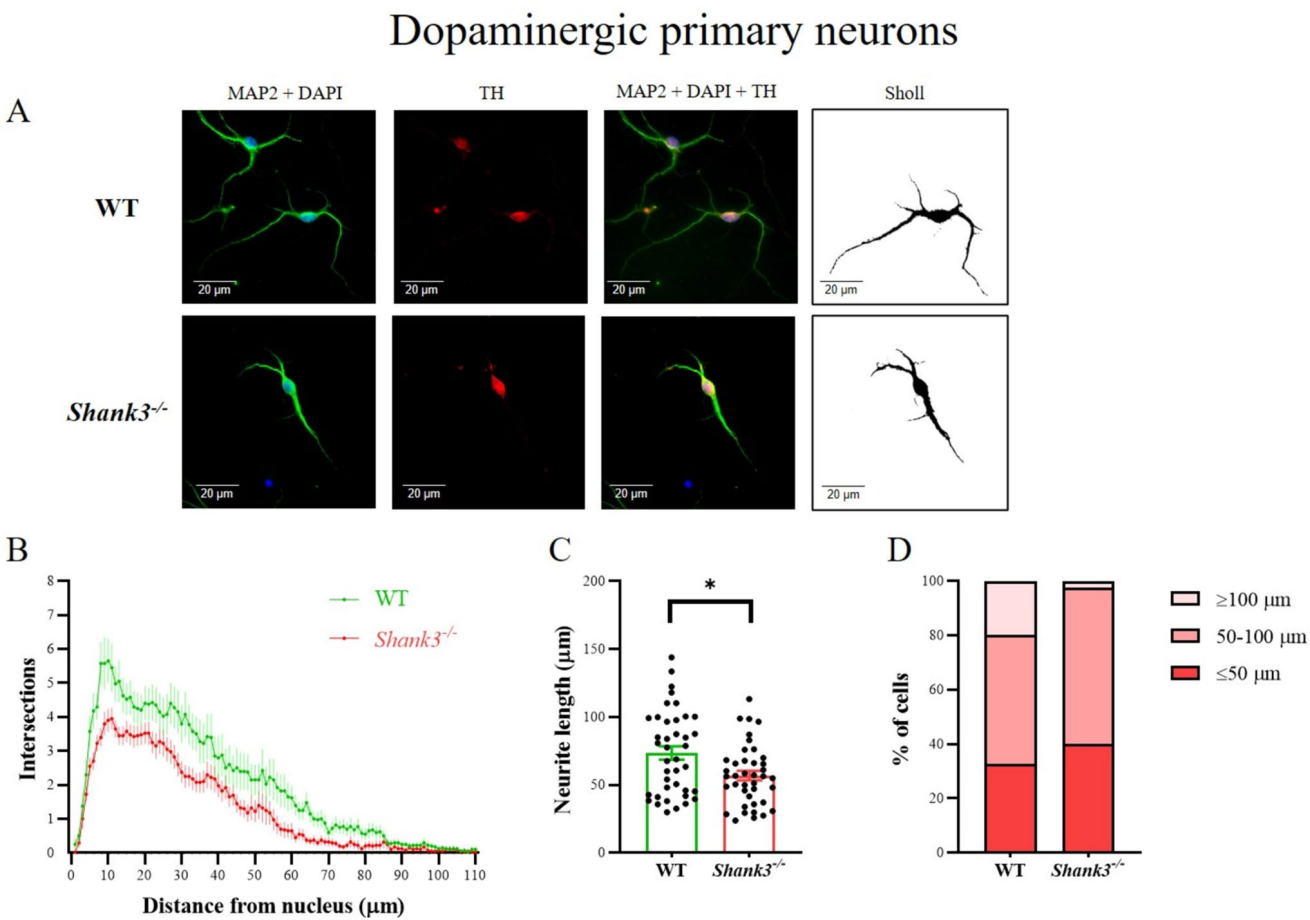
Dendritic arborization in primary dopaminergic neurons isolated from the ventral tegmentum of the midbrain in neonatal WT and *Shank3*-deficient mice. Neurons were incubated in vitro for 9 days and then stained. Overlapping microtubule-associated protein 2 (MAP2) and tyrosine hydroxylase (TH) positive fluorescent signal was considered a marker of the dopaminergic neurons (**A**). The arborization of the dendritic tree was assessed using Sholl analysis. The number of dendrite intersections with various concentric circles is represented in graph (**B**), longest neurites (**C**) and the number of neurons with the longest neurite (**D**) as mean ± SE (*n* = 40 neurons per group isolated from 4 (WT) or 3 (*Shank3*^−*/*−^) mice). Statistical differences between groups were determined by two-way ANOVA (factor genotype *F*_(1, 8658)_ = 264.8; *p* < 0.001; factor arborization *F*_(110, 8658)_ = 39.69; *p* < 0.001) for Sholl analysis or Mann–Whitney test (*p < 0.05) for the longest neurite. WT, wild type

Primary neuronal cell cultures were derived from the ventral tegmentum of the midbrain for the purpose of examining dopaminergic neuron morphology. TH-positive neurons were specifically stained (Fig. 1A). Sholl analysis revealed significant differences in the number and branching of neurites in dopaminergic neurons isolated from the brain of *Shank3*-deficient compared to WT mice. Overall, dopaminergic neurons isolated from *Shank3*-deficient mice had a significantly (two-way ANOVA, factor genotype *F*_(1, 8658)_ = 264.8; *p* < 0.001; factor arborization *F*_(110, 8658)_ = 39.69; *p* < 0.001) reduced number of neurites that were also shorter in length (Fig. 1B). Furthermore, the length of the longest neurite was significantly shorter (*p* < 0.05, Mann–Whitney) in *Shank3*-deficient compared to WT mice (Fig. 1C). We also found that the number of neurons with the neurites over 100 µm was reduced in *Shank3*-deficient (2.5% of cells) compared to WT mice (20% of cells, Fig. 1D). Similar trends were also observed in the case of the number of cells with short neurites.

**Fig. 2:**
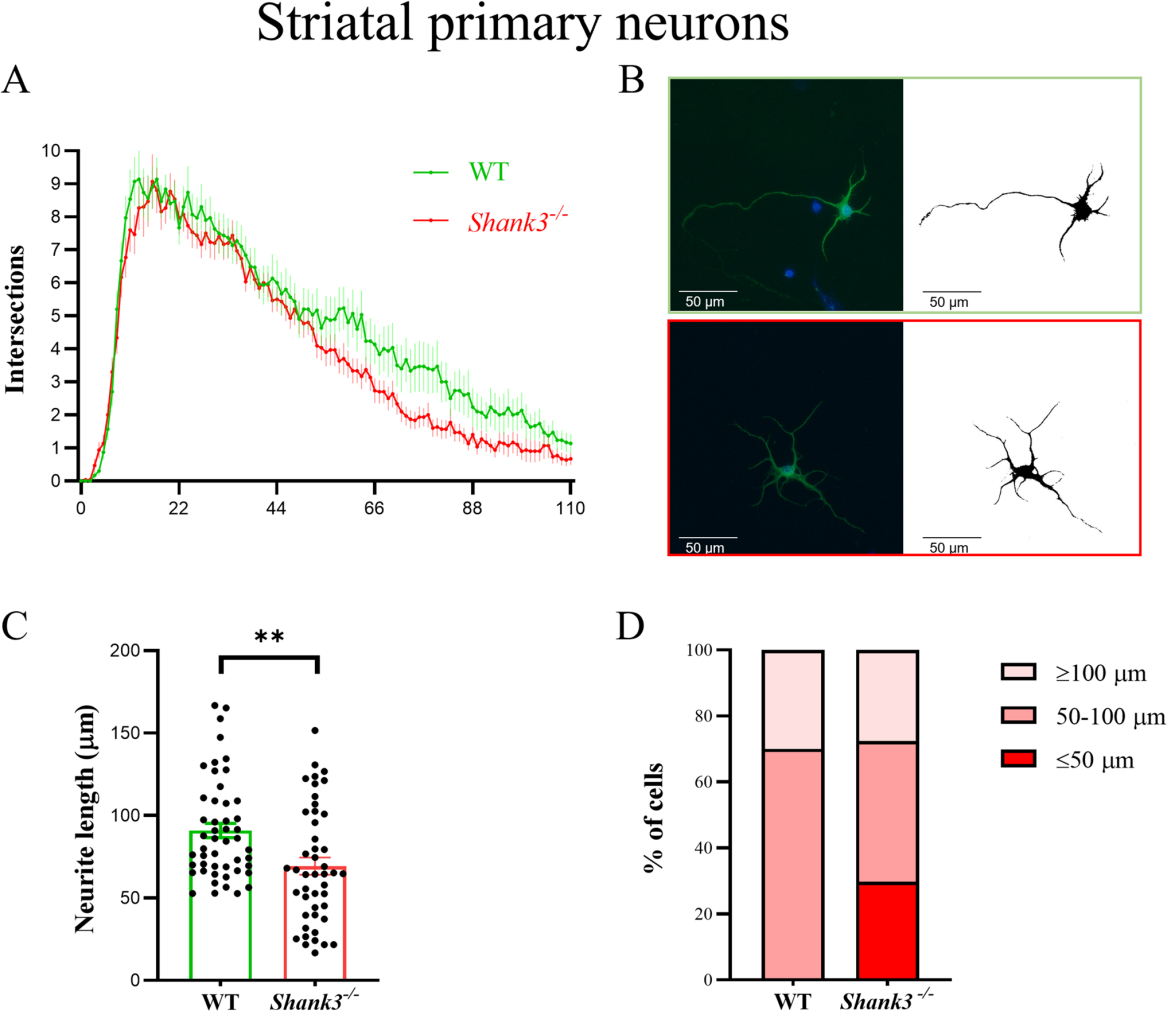
Dendritic arborization in primary striatal neurons isolated from WT and *Shank3*-deficient mice. Neurons were incubated in vitro for 9 days and then stained. Microtubule-associated protein 2 (MAP2) was used to identify neurons (**B**). Cell nuclei were stained with DAPI (blue). The arborization of the dendritic tree was assessed using Sholl analysis. The number of dendrite intersections with various concentric circles is represented in graph (**A**), longest neurites (**C**) and the number of neurons with the longest neurite (**D**) as mean ± SE (*n* = 30 per group in Sholl analysis, *n* = 50 per group in longest neurite evaluation isolated from 4 (WT) or 3 (*Shank3*^−*/*−^) mice). Statistical differences between groups were determined by two-way ANOVA (factor genotype *F*_(1, 6438)_ = 112.1; *p* < 0.001; factor arborization *F*_(110, 6438)_ = 53.83; *p* < 0.001) for Sholl analysis or Mann–Whitney test (**﻿*p* < 0.01) for the longest neurite. WT, wild type

Next, we analyzed the morphology of striatal neurons. We focused on MAP2-positive cells and assessed the number and length of neurites for all these neurons (Fig. 2). We found that neurons isolated from *Shank3*-deficient mice had a significantly reduced number of neurites, which were also shorter in length (two-way ANOVA, factor genotype *F*_(1, 6438)_ = 112.1; *p* < 0.001; factor arborization *F*_(110, 6438)_ = 53.83; *p* < 0.001). Furthermore, the length of the longest neurite was similarly as in the case of tegmental neurons, significantly shorter (Mann–Whitney test, *p* < 0.01) in the group from *Shank3*-deficient mice.

**Fig. 3:**
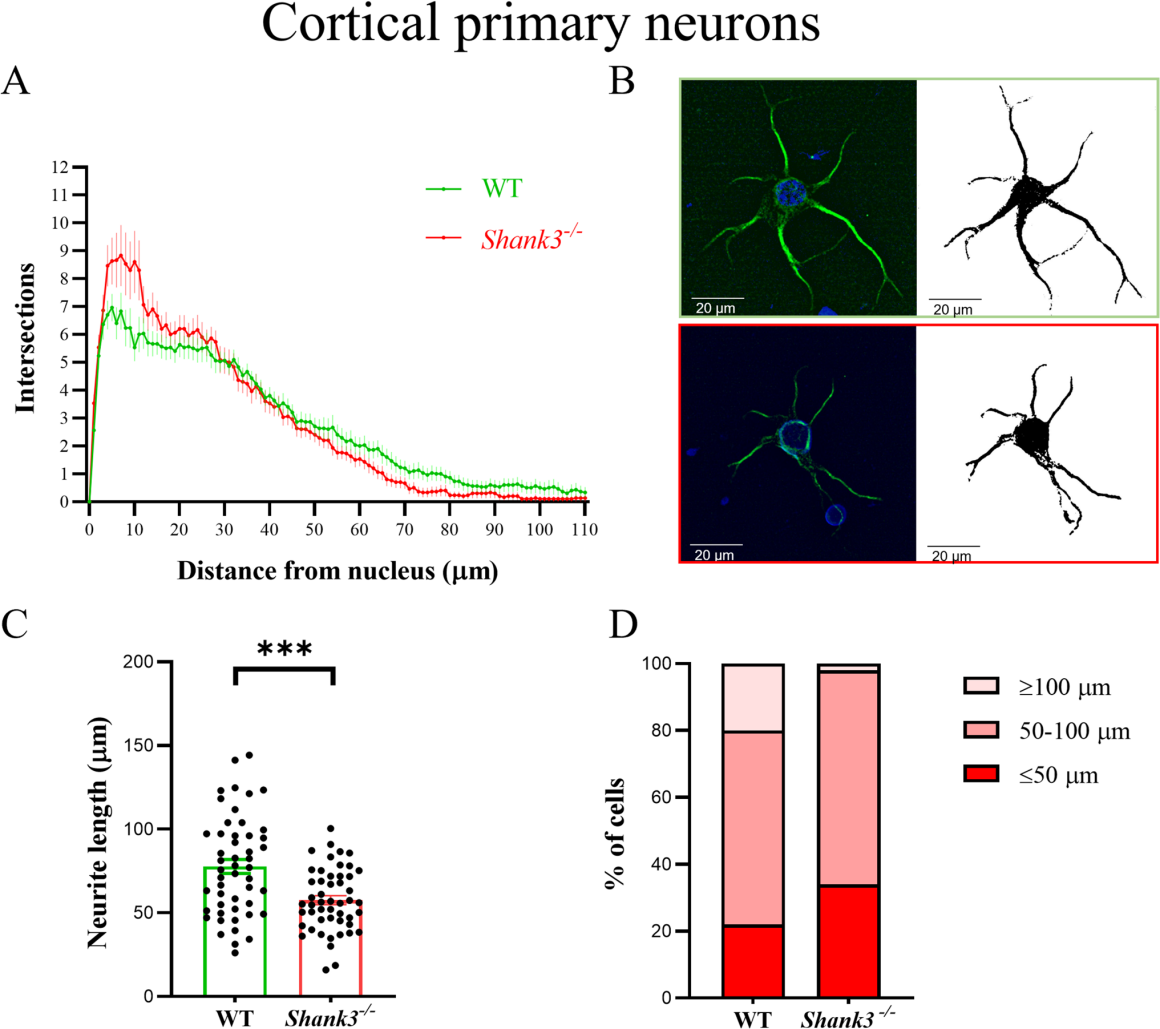
Dendritic arborization in primary cortical neurons isolated from WT and *Shank3*-deficient mice. Neurons were incubated in vitro for 9 days and then stained. Microtubule-associated protein 2 (MAP2) was used to identify neurons (**B**). Cell nuclei were stained with DAPI (blue). The arborization of the dendritic tree was assessed using Sholl analysis. The number of dendrite intersections with various concentric circles is represented in graph (**A**), longest neurites (**C**) and the number of neurons with the longest neurite (**D**) as mean ± SE (*n* = 30 per group in Sholl analysis, *n* = 50 per group in longest neurite evaluation isolated from 4 (WT) or 3 (*Shank3*^−*/*−^) mice). Statistical differences between groups were determined by two-way ANOVA in factors arborization *F*_(110, 6438)_ = 82.13; *p* < 0.001 and interaction *F*_(110, 6438)_ = 2.084; *p* < 0.001. Furthermore, the length of the longest neurite was significantly shorter (﻿﻿***p < 0.001, Mann–Whitney) in *Shank3*-deficient mice. WT, wild type

We did not find overall differences in the shape of cortical neurons between *Shank3*-deficient and WT mice, despite observing significant changes in individual neurons (Fig. 3). More specifically, two-way ANOVA revealed significant differences in factors interaction *F*_(110, 6438)_ = 2.084; *p* < 0.001 and arborization *F*_(110, 6438)_ = 82.13; *p* < 0.001, with a significantly higher number of short neurites in cortical neurons isolated from *Shank3*-deficient compared to WT mice. The Sidak post hoc test revealed significant differences in the number of intersections in the interval within 6–11 µm from the cell nucleus. We also found significantly shortened the longest neurites in *Shank3*-deficient mice (*p* < 0.001, Mann–Whitney).

**Fig. 4:**
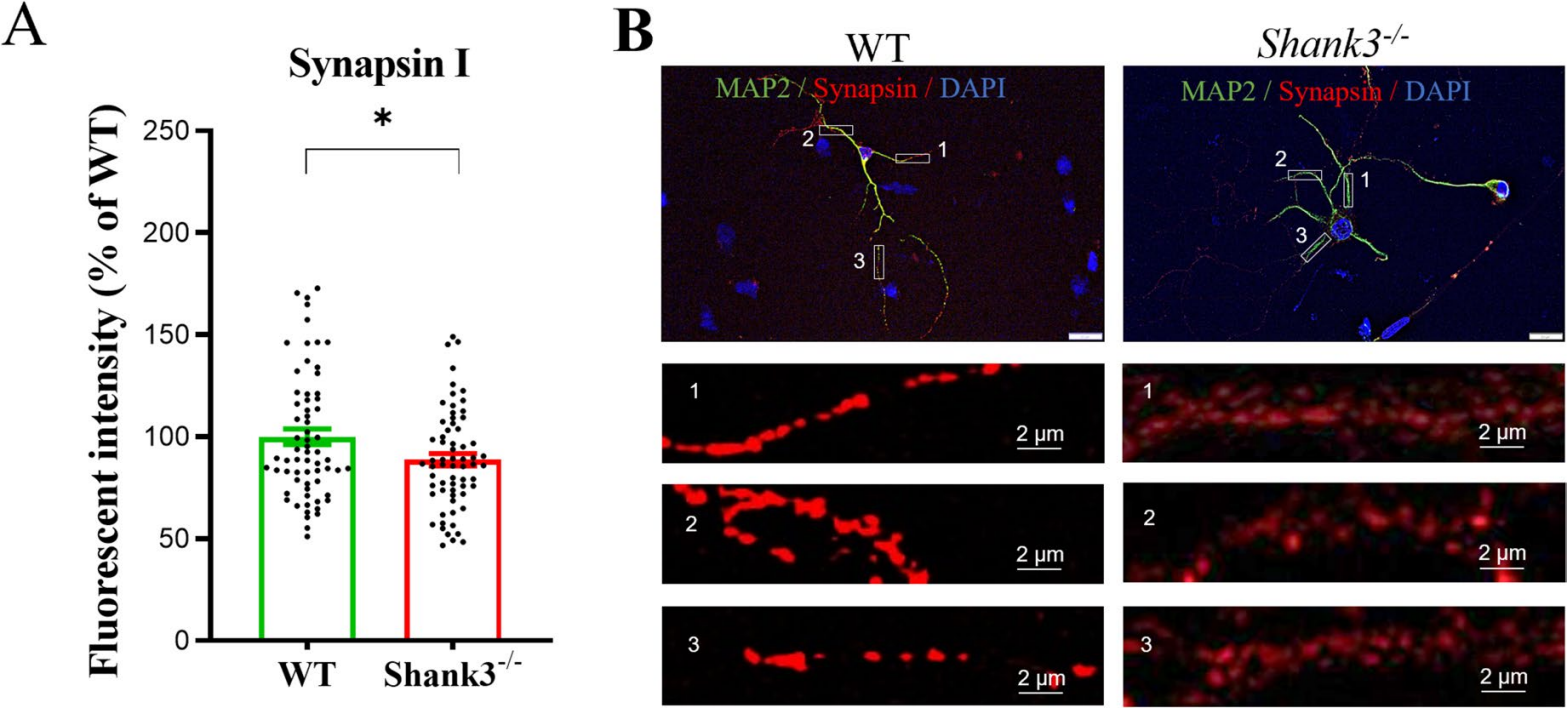
Synapsin I immunofluorescence signal in primary cortical neurons isolated from WT and *Shank3*-deficient mice. Neurons were incubated in vitro for 9 days and then stained. Microtubule-associated protein 2 (MAP2) was used to identify neurons. Cell nuclei were stained with DAPI. Quantitative assessment of Synapsin I immunofluorescence signal was performed in three regions of interest (ROI) per cell (*n* = 65 cells isolated from 4 (WT) or 3 (*Shank3*^−*/*−^) mice), with the size of 20 × 5 µm. Data are represented as a percentage of the control group signal (**A**). Representative neurons (scale bar = 20 µm) isolated from WT and Shank3-deficient mice (**B**); *p < 0.05, Student’s *t*-test. WT, wild type

Immunofluorescence analysis (Fig. 4) revealed a significantly (*T* = 2.27; df = 128; *p* < 0.05) reduced quantitative signal for Synapsin I in the cortical neurons isolated from *Shank3*-deficient compared to WT mice.

### Shank3 Deficiency Changes the Gene Expression of Dopaminergic Receptors but Does Not Affect Synaptic Molecules in the Cortex and Striatum

**Fig. 5:**
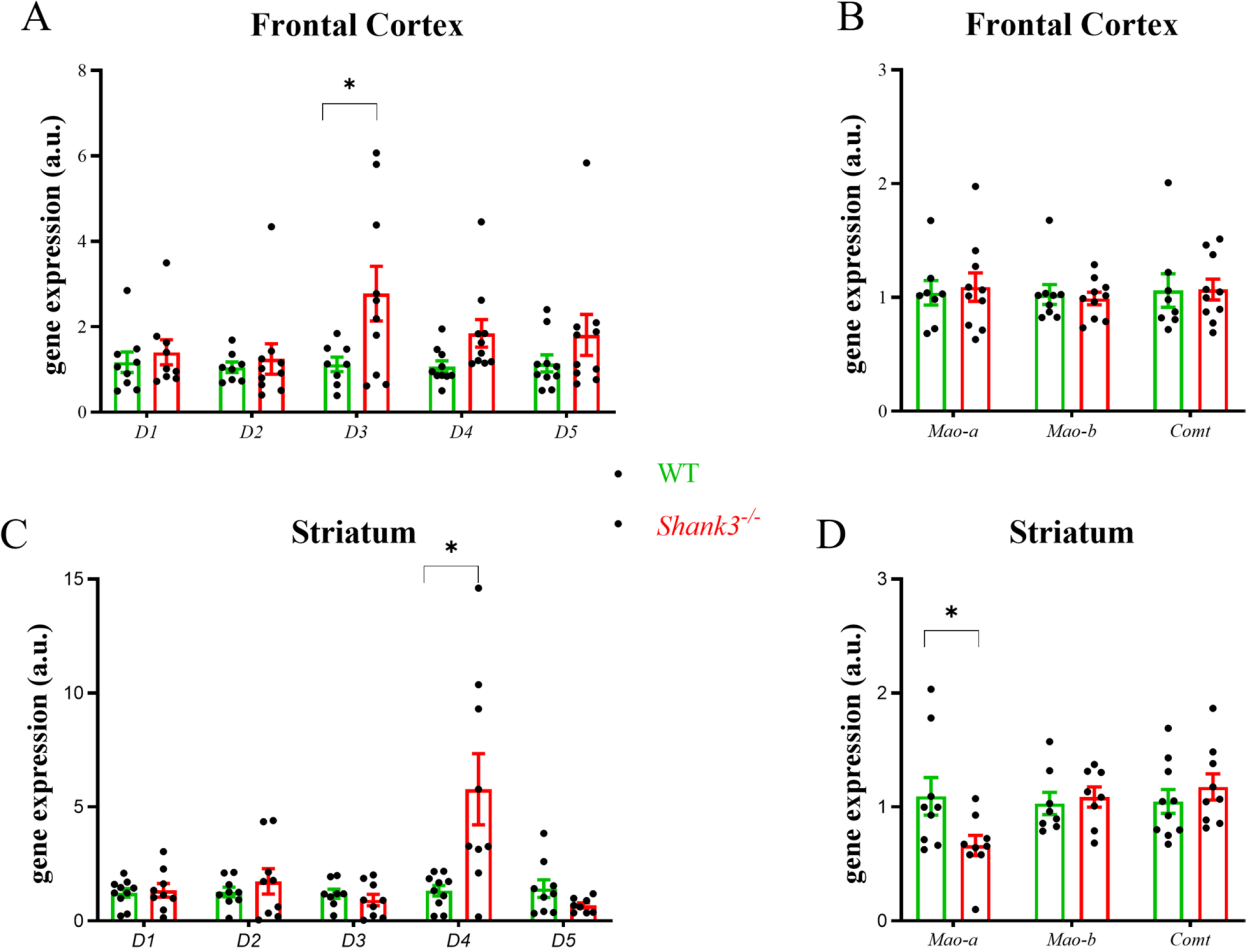
Changes of gene expressions of dopamine receptors and enzymes involved in dopamine metabolism. Frontal cortex **(A**, **B)** and striatum **(C**, **D)** were isolated from *Shank3*-deficient and WT 21-day old male mice. Figures represent relative changes compared to WT group calculated by the 2^−^^∆∆CT^ method. Glyceraldehyde 3-phosphate dehydrogenase was selected as the reference gene. Column scatter dot plot represents values of individual samples and bar shows means ± SE (*n* = 8–9). Significantly different values are marked with *p < 0.05, Student’s *t*-test. WT, wild type; Comt, catechol-O-methyltransferase; D1-D5, dopamine receptors (1–5); Mao-a, b, monoamine oxidase

To understand whether neurite outgrowth and Synapsin I alterations observed in vitro can be manifested in dopaminergic projection areas (striatum, frontal cortex), we evaluated the gene expression of selected synaptic proteins, dopaminergic receptors, and dopamine metabolism enzymes. In contrast to nonsignificant differences in presynaptic and postsynaptic proteins (Table 4), we observed significant changes in the profile of dopaminergic receptors in *Shank3*-deficient mice (Fig. 5). The gene expression of D3 receptor subtype was significantly increased in the frontal cortex (*T* = 2.26; df = 16; *p* < 0.05), and D4 receptor subtype was significantly increased in the striatum (*T* = 2.98; df = 17; *p* < 0.05) in *Shank3*-deficient compared to WT mice. Moreover, the gene expression of *Mao-a* was significantly reduced (*T* = 2.30; df = 16; *p* < 0.05) in the striatum of *Shank3*-deficient compared to WT mice.

**Table 4:** Changes in the gene expression of selected synaptic protein in the frontal cortex and striatum isolated from wild type (WT) and *Shank3*^−*/*−^ mice. Relative gene expression levels were calculated by using the Livak method [26]. Results are presented as means ± SE (*n* = 8–9). Group differences were nonsignificant, but a trend was observed in *Sv2a* (*T* = 2.06; df = 15; *p* = 0.056). *Sv2a*, synaptic vesicle glycoprotein 2A; *Nlgn 1–3*, neuroligin (1–3); *Psd95*, postsynaptic density protein 95

	Frontal cortex		Striatum	
	WT	*Shank3*^−*/*−^	WT	*Shank3*^−*/*−^
*Synapsin I*	1.05 ± 0.15	1.39 ± 0.15	1.13 ± 0.19	0.84 ± 0.13
*Synapsin II*	1.07 ± 0.14	1.20 ± 0.15	1.23 ± 0.26	1.33 ± 0.27
*Synaptophysin*	1.14 ± 0.19	1.39 ± 0.31	1.38 ± 0.31	2.14 ± 0.88
*Sv2a*	1.03 ± 0.08	0.81 ± 0.05	1.29 ± 0.33	0.90 ± 0.18
*Nlgn1*	1.40 ± 0.39	1.90 ± 0.86	1.42 ± 0.46	1.17 ± 0.32
*Nlgn2*	1.06 ± 0.12	2.51 ± 1.16	1.03 ± 0.07	2.73 ± 1.24
*Nlgn3*	1.16 ± 0.37	4.24 ± 1.69	1.34 ± 0.32	1.19 ± 0.23
*Psd95*	1.10 ± 0.18	1.87 ± 0.81	1.13 ± 0.17	1.60 ± 0.33

## Discussion

In the present study, we found impaired neurite outgrowth and dendritic arborization in primary cultures of dopaminergic neurons isolated from *Shank3*-deficient mice. Our new data observed in tegmental and striatal neurons are consistent with previously observed abnormal morphology of hippocampal neurons in the conditions of *Shank3* deficiency [16]. In the present study, we also observed a decrease in Synapsin I immunofluorescence signal in the cortical neurons isolated from *Shank3*-deficient mice, although arborization changes were less severe. Importantly, we confirmed the deficit in the length of the longest neurite in the primary cortical neurons isolated from *Shank3*-deficient mice. No changes in the gene expression of synaptic proteins were observed in the striatum and frontal cortex of *Shank3*-deficient mice, but an altered gene expression profile of dopaminergic receptors, including reduced *Mao-a* expression, was found.

We clearly confirmed that *Shank3* deficiency is accompanied by significant changes in the morphology of neurons isolated from dopaminergic brain regions. In the case of tegmentum, we focused on TH-positive cells, which represent a well-established embryonal and/or postnatal model of dopamine-producing neurons [34–36]. Although at the macroscopic level we could not directly isolate neurons from the “ventral tegmental area” (VTA) itself, the region we obtained from our primary neuron cell cultures corresponds to the major source of midbrain dopaminergic neurons. Our findings showing a reduced number of neurites that are also shorter in length in *Shank3*-deficient compared to WT mice are new in this model. These data also point to the fact that changes in the growth of neurites of dopaminergic neurons may contribute to the structural abnormalities of autism. In fact, this corresponds to the theory that structural defects in single axons and associated dopaminergic pathways could play a role in autism pathogenesis [37, 38]. Moreover, in the different autism-like models with prenatal valproate exposure, hypertrophy and changes in dendritic spines in the tissue of nucleus accumbens were observed at different stages of brain development [39]. Another study reported that exposure to p-cresol, a metabolite implicated in autism, led to decreased dendritic arborization in neuronal cell cultures [40]. We did not distinguish between dendrites and axons; nevertheless, given that the length of the longest neurite was significantly shorter in dopaminergic neurons isolated from *Shank3*-deficient mice, we conclude that it is likely that the axons of neurons also grow to a shorter length. These changes could be reflected in dopaminergic projection areas, which, however, requires further studies, especially in vivo.

In this study, we found a decrease in the neurite (dendritic) arborization of neurons isolated from the striatum of *Shank3*-deficient mice, and conversely, we observed a significantly higher number of short neurites in cortical neurons isolated from *Shank3*-deficient compared to WT mice. This result points to differences in the morphology of striatal and cortical neurons in conditions of *Shank3* deficiency, which could be more pronounced at the structural level. In this study, morphological changes were observed on DIV 9, but other stages of neurite outgrowth or synaptogenesis could be differently affected. It is also important to note that neurons at DIV9 are not fully matured, which may be an additional factor contributing to the differences observed in autistic conditions. Nevertheless, neuron morphology changes in the striatum of *Shank3*-deficient mice have already been described in the past. Peça et al. [10] found that *Shank3B*^*−/−*^ mice displayed a significant reduction in spine density with neuronal hypertrophy measured by an increase in complexity of dendritic arbores. Another study suggested that deleting exons 13–16 from the *Shank3* gene leads to a decrease in dendritic spine density and reduces spine head diameter with impairment in corticostriatal synaptic transmission [18]. These authors also point to the fact that SHANK3 protein isoforms are differentially expressed by direct and indirect pathway spiny projection neurons in the striatum. Given the multiple isoforms and neuron-specific expression patterns of *Shank3*, it is necessary to be careful when interpreting our results.

We found a decrease in Synapsin I immunofluorescence in primary cortical neurons isolated from *Shank3*-deficient mice, but no changes in gene expression of other presynaptic or postsynaptic proteins either in the prefrontal cortex or striatum were found. We observed a tendency for a decrease in *Sv2a* gene expression in the cortical area in *Shank3*-deficient mice at the margin of statistical significance. These results are in contrast with the findings of Peca et al. [10], who found reduced protein levels of glutamate receptor subunits GluR2, NR2A, and NR2B, as well as postsynaptic proteins SAPAP3, Homer, and PSD93 in the striatum in *Shank3*-deficient mice. Possible subtle changes in our homogenates might be masked by whole tissue processing. Another factor to consider could be the age of the animals from which the tissues were isolated; in our study, we used immature 21-day-old mice. The brain is still developing and maturing at the postnatal day 21 but has progressed beyond early infancy. In this context, on postnatal day 21, other studies have observed dendritic pathology in the brain and behavioral changes in autism-like mouse models [41, 42]. Regardless, our findings support the idea that presynaptic proteins, potentially due to changes in neurite outgrowth, may play a role in brain dopaminergic projection areas in the autistic conditions, a concept also suggested by other animal and human studies [43, 44]. Although interpretation requires caution, mRNA and protein synaptic levels may differ and depend on the developmental stage. Previous findings concerning the alterations in dopaminergic areas of the brain in *Shank3* deficiency can also be confirmed by present data demonstrating increased gene expression of the *D3* receptor in the frontal cortex and the *D4* receptor in the striatum in *Shank3*-deficient mice. Reduced *Mao-a* expression in the striatum could affect dopamine metabolism by decreasing the breakdown of dopamine. Similar findings were observed by others, namely, increased D2 receptor binding density in the ventral striatum in *Shank3*-deficient mice, and a similar trend was observed in the *Fmr1* mouse model of autism [3]. A more complex study in autistic subjects, using a D2/D3 dopamine receptor antagonist, has shown decreased striatal dopamine release, which authors interpreted as supporting neurobiological evidence of impaired social motivation in ASD [45].

Our findings in the *Shank3*-deficient mouse model of autism, together with previous data, suggest that morphological changes in dopaminergic neurons and alterations in neurite growth can influence projection areas, synaptic protein expression, and dopamine receptor levels. These changes could potentially contribute to the long-term development of autistic symptoms.

## Data Availability

Data are available upon reasonable request.
